# The 4 Mountains Test: A Short Test of Spatial Memory with High Sensitivity for the Diagnosis of Pre-dementia Alzheimer's Disease

**DOI:** 10.3791/54454

**Published:** 2016-10-13

**Authors:** Dennis Chan, Laura Marie Gallaher, Kuven Moodley, Ludovico Minati, Neil Burgess, Tom Hartley

**Affiliations:** ^1^Department of Clinical Neurosciences, University of Cambridge; ^2^Clinical Imaging Sciences Centre, Brighton and Sussex Medical School; ^3^U.O. Direzione Scientifica, Fondazione IRCCS Istituto Neurologico Carlo Besta; ^4^Institute of Cognitive Neuroscience, University College London; ^5^Department of Psychology, University of York

**Keywords:** Behavior, Issue 116, Alzheimer's disease, mild cognitive impairment, spatial memory, hippocampus, 4 Mountains Test, pre-dementia Alzheimer's disease

## Abstract

This protocol describes the administration of the 4 Mountains Test (4MT), a short test of spatial memory, in which memory for the topographical layout of four mountains within a computer-generated landscape is tested using a delayed match-to-sample paradigm. Allocentric spatial memory is assessed by altering the viewpoint, colors and textures between the initially presented and target images.

Allocentric spatial memory is a key function of the hippocampus, one of the earliest brain regions to be affected in Alzheimer's disease (AD) and impairment of hippocampal function predates the onset of dementia. It was hypothesized that performance on the 4MT would aid the diagnosis of predementia AD, which manifests clinically as Mild Cognitive Impairment (MCI).

The 4MT was applied to patients with MCI, stratified further based on cerebrospinal fluid (CSF) AD biomarker status (10 MCI biomarker positive, 9 MCI biomarker negative), and with mild AD dementia, as well as healthy controls. Comparator tests included tests of episodic memory and attention widely accepted as sensitive measures of early AD. Behavioral data were correlated with quantitative MRI measures of the hippocampus, precuneus and posterior cingulate gyrus.

4MT scores were significantly different between the two MCI groups (p = 0.001), with a test score of ≤8/15 associated with 100% sensitivity and 78% specificity for the classification of MCI with positive AD biomarkers, *i.e.,* predementia AD. 4MT test scores correlated with hippocampal volume (r = 0.42) and cortical thickness of the precuneus (r = 0.55).

In conclusion, the 4MT is effective in identifying the early stages of AD. The short duration, easy application and scoring, and favorable psychometric properties of the 4MT fulfil the need for a simple but accurate diagnostic test for predementia AD.

**Figure:**
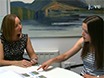


## Introduction

Alzheimer's Disease (AD) is the most common cause of dementia and it is now understood that predementia stages of AD exist in the form of a clinically silent "presymptomatic" stage. A symptomatic / "prodromal" stage, manifests itself as mild cognitive impairment (MCI), in which individuals exhibit cognitive decline (typically memory impairment) but retain functional independence and preserved activities of daily living^1^. This clinical reconsideration of AD is reflected in the current sets of diagnostic criteria for AD^2,3^.

Diagnosis of AD in its predementia stages is critically important, not just for provision of best clinical care but also in light of the anticipated arrival of disease-modifying treatments for AD, given that such treatments are likely to be most effective if applied at earlier stages of the disease. This is illustrated by the recent clinical trials of the anti-amyloid drug solanezumab; while initial results failed to show any treatment effect, reevaluation of data on the subgroup of trial patients with milder disease found a significant effect on the trial's cognitive endpoints^4^.

However, MCI due to AD is very difficult to distinguish clinically from other causes of MCI, which also include nonneurodegenerative disorders such as anxiety. There is a need therefore for a test that is not only sensitive to AD-related dementia but also noninvasive and suitable for widespread use in routine clinical diagnostic practice. The latter requirement is crucial in view of the high prevalence of MCI in the ageing population, with an estimated prevalence of 5 - 20% in the UK population aged over 65 years^5, ^many of whom will attend nonspecialist, community-based, memory clinics.

Current tests do not meet this need. Tests used to detect AD-related dementia (such as MRI scanning to determine whole brain or hippocampal atrophy) have reduced sensitivity when applied to the pre-dementia stages of AD^6^. The Mini Mental State Examination (MMSE)^7^ is the most widely used cognitive test in memory clinics but is insensitive, nonspecific and has poor predictive ability^8^. Biomarker-based tests, such as amyloid-PET or CSF studies of amyloid/tau, are good predictors of conversion to dementia^9^ but are invasive and expensive, and their restricted availability to select specialist clinics precludes their usage in routine clinical practice.

The 4 Mountains Test (4MT)^10^ may meet this need. The 4MT is a short test of working allocentric spatial memory (that assesses the ability of the participant to recall the spatial configuration of a series of computer-generated landscapes from a shifted viewpoint), designed to reflect the role of the hippocampus in spatial cognition. The scientific rationale for the use of such a test is founded on two principles, both supported by extensive research. The first is that the hippocampus and the related medial temporal lobe (MTL) structures are affected from the earliest stages of AD. Evidence for this is obtained from neuropathological studies of AD, which have shown that neurodegeneration is observed initially within the MTL, with involvement first of the entorhinal cortex and subsequently, the hippocampus^11, 12^. Severe neuronal loss in these regions is present even at the earliest stages of clinically-evident AD^13^. The second principle is that the hippocampus is critically involved in spatial memory. This originates from the initial demonstration of place-related firing activity of hippocampal neurons in freely moving rats^14^, which led to the "cognitive map" theory of hippocampal function^15^. Subsequent work has indicated that the human hippocampus has a similar role, with place-related activity observed during presurgical recordings from the hippocampal neurons of patients with temporal lobe epilepsy^16^, and with activation of the hippocampus in functional imaging studies during tasks involving spatial memory^17^.

The 4MT task and stimuli are described in Hartley *et al*. (2007)^10^. The same stimuli have been used in all later studies published to date^18-21^.

Each item in the 4MT is composed of 5 images of computer-generated landscapes as shown in **Figure 1**. Participants view a sample image and are then required to choose a target image from 4 alternatives, which shows the same place from a different viewpoint. The remaining 3 images are foils depicting landscapes whose topography differs systematically from the target landscape.


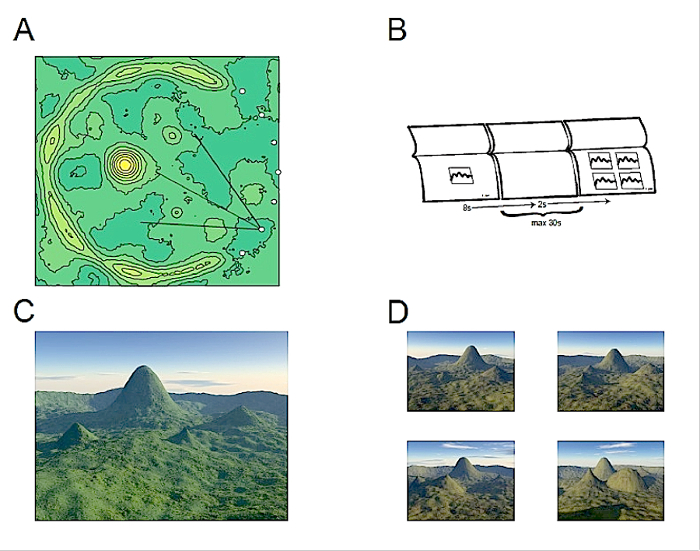
**Figure 1. The Four Mountains Test (4MT)**. **(A)** All 4MT stimuli are based on computer-generated heightfields containing 4 mountains as illustrated by a contour map for one example (see text for further details). Images are rendered using a virtual camera placed at one of the indicated 7 locations. **(B)** Participants see a sample image which they study before seeing four different images (one target showing the same place from a different view point, and 3 foils showing different places). Their task is to identify the target. **(C)** Example of a sample image. **(D)** Corresponding target and foil images (the target is seen top-left). Note that all images are shown at the same scale in the test, and that viewpoint and other nonspatial features are systematically varied between sample and test images. Please click here to view a larger version of this figure.

Each landscape is made up of similar topographical features: the ground plane with small scale undulations, a semicircular mountain range (defining the horizon in each image), and 4 prominent mountains of varying shapes and sizes. An example is shown as a contour map in **Figure 1A**. Stimuli are selected from a large number of landscapes, created such that each has an unambiguously distinct global topography within which individual local features are shared between items and between target and foil landscapes. Landscapes used to generate foil images are created by varying the size shape and location of mountains in the sample/target landscape. This provides a set of foils sharing (to a similar degree across items) local topographical features, such that nonspatial strategies based on memory for local features would be ineffective. Sample and target images are rendered using the same topography but different camera locations. The aim of the viewpoint change is to encourage allocentric spatial strategies (exploiting spatial representations which are known to exist within the hippocampal formation, see Hartley *et al*., 2014 for a recent review^22^) and to discourage strategies based on egocentric or visual representations (which are known to exist outside the hippocampal formation, see Burgess, Jeffery & O'Keefe, 1999^23^). The latter type of information is disrupted by the unpredictable shift of viewpoint, especially since several topographical features are common to both target and foil landscapes. To further discourage visual pattern matching strategies, the lighting, landscape colors and textures and weather conditions are varied between the sample and the test images. Each item thus comprises a sample image with one set of nonspatial features, a target image depicting the same landscape from a different viewpoint and different nonspatial features and 3 foil images with distinct topography but sharing nonspatial features with the target.

Following the demonstration that 4MT spatial memory performance (Place Memory; PM) was selectively impaired in patients with focal hippocampal damage, while spatial perception (Place Perception; PP), nonspatial memory and nonspatial perception were relatively spared^10^, this test was applied to patients with dementia. Results from separate research groups showed that the 4MT could differentiate patients with AD-related dementia, not only from age-matched control subjects but also from patients with other dementia-causing disorders^18, 19^.

More recently, the 4MT has been shown to discriminate MCI patients with and without biomarker evidence of underlying AD, illustrating its potential utility as a clinical test for predementia AD^20^. As part of the same study, the 4MT was successfully applied to an MCI patient cohort recruited from Italian memory clinics, demonstrating the utility of this test, the design of which is language-independent, in different clinical and cultural settings.

This paper describes the 4MT methodology and summarizes the results of the study on MCI patients, which are published in full in the paper by Moodley *et al*. (2015)^20^. In this study, performance on the 4MT was compared with structural measurements of key brain regions involved in spatial processing, namely the hippocampus, precuneus and posterior cingulate gyrus.

## Protocol

### 1. Participant selection criteria

1. Select individuals who are not color-blind and have normal or corrected-to-normal vision.

### 2.Test Preparation

1. Seat the patient or control participant in a quiet room.
2. Ensure the patient or control participant has glasses to correct their vision, if applicable.

### 3. Practice Test

1. Instruct the participant as follows: *"In this test you will see a picture of a mountain landscape which you should study carefully. That picture will be followed by four similar landscapes seen from different points of view and under different conditions of lighting or weather." "One of the four pictures shows exactly the same place as in the previous picture, although it will be shown from a slightly different viewpoint and conditions of lighting or weather. Your task is to identify which of the four pictures shows the same place as the one you have just seen." "Focus on the layout of the scene (the shape and arrangement of mountains and other geographical features). "Which picture shows the place in the previous picture?"*
2. Instruct the participant to complete 3 practice items.
  1. Provide verbal feedback on these items where necessary, drawing participants' attention to relevant features of the stimuli.
  2. During the practice phase, instruct the participant to ask for clarification if they are unsure about any aspect of the task, and reinforce the original instructions as necessary, before proceeding to the test items.

### 4. Main Test

1. Inform the participant that they will be given the main test, including how many questions to expect and how much time they will have on each. For example: *"Now you're going to do the main test. There are 15 questions in total, and they are just like the practice ones you have just done. I'll give you a short time to study each picture, and then you have about 20 sec within which to choose your answer."*
2. Present the test items and record responses, allowing the participant a total of 30 sec for each item. Turn the pages of the booklet to control the timing of the stimulus presentation and responses.
3. Start timing when the sample image is presented.
4. Show the sample image for 8 sec, then turn to a blank page (1 sec), then turn to the page containing the response images (1 sec) and then show the response images for up to 20 sec, or until the participant indicates their selection.
5. Ask the participant to indicate their response by pointing to the selected image. Stop timing when the participant and has made their selection.
6. Record the participant's response and the time taken to make that response on the corresponding response sheet, giving no feedback on whether the response is correct or not.

### 5.Test Scoring

1. After the testing session, score the total number of correct responses (a simple raw total).

## Representative Results

The study was performed in accordance with the Declaration of Helsinki. All participants gave written, informed consent. Ethics approval was obtained from the UK Research Ethics Committee South East Coast and from Brighton and Sussex University Hospitals NHS Trust (references 10/H1107/23 and 13/LO/0277, respectively).

21 patients with MCI were recruited form the Cognitive Disorders Clinic at Hurstwood Park Neurological Centre, Haywards Heath, West Sussex, UK. MCI was diagnosed according to internationally recognized criteria^1^, which specify I) a subjective report of cognitive decline, corroborated by an informant II) objective evidence of cognitive impairment on formal testing III) absence of dementia and IV) preserved activities of daily living and functional independence.

Objective cognitive testing was undertaken using either the Addenbrooke's Cognitive Examination-Revised^24^ or the Queen Square Screening Test for Cognitive Deficits (EK Warrington 2003) in combination with the Mini Mental State Examination (MMSE)^7^. As part of the clinical diagnostic workup, patients underwent clinical and laboratory assessments to exclude potentially treatable causes of cognitive decline, such as vitamin B12 deficiency or thyroid dysfunction. The presence of significant cerebrovascular disease was a core exclusion criterion, as evidenced by significant vascular lesion load on imaging (the presence of cortical infarcts, extensive and/or confluent White Matter Hyperintensities (WMH) and WMH >10mm diameter), and/or a Hachinski Ischaemic Score >4^25^. Patient data were compared with that from age-matched healthy controls (HC) without a history of cognitive impairment and with 11 patients with mild AD-related dementia, diagnosed according to the McKhann criteria^26^.

The MCI patient group was split into MCI biomarker positive (MCI+ve) and MCI biomarker negative (MCI-ve) subgroups on the basis of testing for CSF biomarker evidence of underlying AD pathology, *i.e.*, CSF β-amyloid_1-42 _and tau levels. Biomarker positive/negative status was determined using updated cut-off scores^27^. The detection of positive CSF biomarkers in MCI patients (*i.e.*, the MCI+ve subgroup) would fulfil diagnostic criteria for predementia AD, termed variously as prodromal AD^2^ or MCI due to AD^3^. Two MCI patients did not undergo CSF testing.

All subjects were tested on a battery of neuropsychological tests, which included testing of the following cognitive domains: premorbid IQ (National Adult Reading Test, Nelson and Willison 1984)^28^, episodic memory (Rey Auditory Verbal Learning Test, RAVLT, Rey 1941)^29^, attention and executive function (Trail Making Test A and B, Reitan 1958)^30^, executive function (lexical and semantic fluency, Benton *et al*. 1994)^31^, working memory (Digit Span, Blackburn and Benton 1957)^32^ and higher visual processing (Object Decision Test from the Visual Object and Space Perception test)^33^

MRI scanning was undertaken on a 1.5T scanner based at the Clinical Imaging Sciences Centre, Brighton and Sussex Medical School, UK. T1-weighted 3D volumetric MRI data were acquired using a magnetization-prepared rapid-acquisition gradient-echo sequence, with 1 x 1 x 1mm^3^ voxel size, TI = 600 msec, TE = 4 msec, TR = 1160 msec. 2 AD patients and 4 MCI patients were unable to undergo MRI scanning. Structural correlations were reported for the remaining participants.

Cortical thickness was measured using the open source FreeSurfer package (Massachusetts General Hospital, Harvard University, Boston MA, USA), which, as detailed elsewhere^34^, involves iterative reconstruction of the white-gray matter interface and pial surface, and subsequent labelling with non-linear morphing to a probabilistic brain atlas. The Desikan probabilistic brain atlas was used^35^, with the posterior cingulate gyrus and precuneus selected as regions of interest (ROIs), reflecting their putative role in spatial cognition and their early involvement in AD^36, 37^.

Total hippocampal volumes were measured using the FSL/FIRST tool (FMRIB, Oxford Centre for Functional Magnetic Resonance Imaging of the Brain, Oxford, UK)^38^. Correlations were not determined for other brain regions, reflecting the study hypothesis. In particular, correlations with frontal brain regions were not calculated since 4MT performance is not impaired in patients with frontotemporal dementia^18, 19^.

All study groups (MCI, AD, HC), and within the MCI biomarker subgroups, were matched in terms of demographics (age, gender, years of education) (**Table 1**).

**Table:** 

**A)**				
	HC	MCI	AD	p
n = 20	n = 21	n = 11
Gender, M:F	7:13	15:06	5:06	0.06
Age, years	62.6 (6.1)	68.1 (8.9)	66.2 (8.9)	0.1
Education, years	12.1 (1.7)	11.7 (1.9)	12.4 (2.2)	0.58
**B)**				
	MCI -ve	MCI +ve	p	
n = 9	n = 10	
Gender, M:F	7:02	8:02	0.67	
Age, years	65 (9.5)	68.1 (6.2)	0.41	
Education, years	11.6 (1.9)	12.1 (2.1)	0.56	
Disease Duration, years	3.8 (0.44)	3.7 (0.82)	0.8	

**Table 1. Demographics of Participants**. Data presented as mean (standard deviation) for **A)** all participants grouped according to cognitive status (HC = Healthy Control, MCI = Mild Cognitive Impairment, AD = Alzheimer's Disease **B)** MCI patients grouped according to CSF AD biomarker status. Reproduced, with permission, from Moodley *et al*. (2015)^20^.

*General neuropsychometric assessment* MCI patients were impaired on tests of episodic memory (RAVLT; delayed recall and recognition memory) and executive function (Trail Making Test A and B). By comparison, and consistent with their diagnostic classification, patients with AD-related dementia were impaired in all cognitive domains (**Table 2**).

**Table:** 

**All participants**
	**HC**	**MCI**	**AD**	**ANOVA**	**HC vs MCI**	**HC vs AD**	**MCI vs AD**
**PP**	4.9	4.3	2.8	F(2,49) = 16.0	p = 0.1	p <0.001	p = 0.001
-0.9	-1.2	-0.8	p <0.001
**PM**	11.1	7.6	4.6	F(2,49) = 32.0	p <0.001	p <0.001	p = 0.004
-2.1	-2.7	-1.3	p <0.001
**MCI participants**		
	**HC**	**MCI-ve**	**MCI+ve**	**AD**	**ANOVA**		
**PP**	4.9	4.9	3.9	2.8	F(3,46) = 13.8		
-0.9	-1.2	-0.9	-0.8	p <0.001		
**PM**	11.1	9.6	5.8	4.6	F(3,46) = 34.3		
-2.1	-1.6	-2.3	-1.3	p <0.001		
**Pairwise comparisons**	
	**HC vs MCI-ve**	**HC vs MCI+ve**	**HC vs AD**	**MCIve- vs MCI+ve**	**MCI-ve vs AD**	**MCI+ve vs AD**	
**PP**	p = 1.0	p = 0.06	p <0.001	p = 0.2	p <0.001	p = 0.09	
**PM**	p = 0.3	p <0.001	p <0.001	p = 0.002	p <0.001	p = 0.6	

**Table 2. Neuropsychometric Data. **Neuropsychometric data for all participants, presented as raw scores, in keeping with the UK clinical practice for reporting of neuropsychometric data, described as mean (standard deviation). NART = National Adult Reading Test. MMSE = Mini Mental State Examination (not performed in control subjects). VOSP-OD = Visual Object and Space Perception battery. RAVLT-DR = Rey Auditory Verbal Learning Test-Delayed Recall (List A). RAVLT-RP = Rey Auditory Verbal Learning Test-Recognition Performance (List A). Reproduced, with permission, from Moodley *et al*. (2015)^20^.

A direct comparison of the MCI subgroups did not reveal any significant differences in the test scores obtained by the MCI-ve and MCI+ve patients, with the exception of the Trail Making Test "B" (**Table 3**). There was no significant difference in episodic memory between the 2 MCI groups (RAVLT; delayed recall and recognition memory).

**Table:** 

	**MCI-ve**	**MCI+ve**	**t(df)**	**Uncorrected p**
**MMSE**	27.6 (0.7)	27.4 (1.3)	0.3 (17)	0.8
**NART**	116.3 (8.0)	109.1 (11.1)	1.5 (16)	0.2
**VOSP**	17 (1.7)	16.4 (2.3)	0.6 (16)	0.5
**RAVLT-DR**	2.8 (2.7)	2.7 (1.8)	0.1 (16)	1
**RAVLT-RP**	0.6 (0.2)	0.6 (0.2)	-0.1 (16)	0.9
**Lexical Fluency**	42.9 (9.2)	36.9 (10.6)	1.3 (16)	0.2
**Semantic Fluency**	28.6 (3.9)	27.9 (6.7)	0.3 (16)	0.8
**Trails A**	37.3 (8.3)	43.8 (16.2)	-1.0 (16)	0.3
**Trails B**	82.6 (24.6)	125.0 (39.0)	-2.7 (16)	0.02
**Digit Span**	6.9 (1.5)	6.3 (0.8)	1.1 (16)	0.3

**Table 3. Neuropsychometric Results for MCI Patients**. Neuropsychometric data for MCI patients, grouped according to CSF AD biomarker status (alpha = 0.004, adjusted for multiple comparisons) presented as raw scores, in keeping with the UK clinical practice for reporting of neuropsychometric data, described as mean (standard deviation). Reproduced, with permission, from Moodley *et al*. (2015)^20^.

*4MT performance* There were significant differences between study groups in terms of performance on the 4MT test (p <0.001, **Table 4**). After correction for multiple comparisons, pairwise group comparisons revealed significant differences between healthy controls (HC) and MCI+ve groups (p <0.001), HC and AD (p <0.001), MCI-ve and AD (p <0.001) and, crucially, between MCI-ve vs MCI+ve groups (p = 0.002). No significant difference in PM test scores was observed between HC and MCI-ve (p = 0.3) or between MCI+ve and AD groups (p = 0.6). **Figure 2** shows individual 4MT scores and the differences in score between study groups.

**Table:** 

**All participants**
	**HC**	**MCI**	**AD**	**ANOVA**	**HC vs MCI**	**HC vs AD**	**MCI vs AD**
**PP**	4.9	4.3	2.8	F(2,49) = 16.0	p = 0.1	p <0.001	p = 0.001
-0.9	-1.2	-0.8	p <0.001
**PM**	11.1	7.6	4.6	F(2,49) = 32.0	p <0.001	p <0.001	p = 0.004
-2.1	-2.7	-1.3	p <0.001
**MCI participants**		
	HC	MCI-ve	MCI+ve	AD	ANOVA		
**PP**	4.9	4.9	3.9	2.8	F(3,46) = 13.8		
-0.9	-1.2	-0.9	-0.8	p <0.001		
**PM**	11.1	9.6	5.8	4.6	F(3,46) = 34.3		
-2.1	-1.6	-2.3	-1.3	p <0.001		
**Pairwise comparisons**	
	HC vs MCI-ve	HC vs MCI+ve	HC vs AD	MCIve- vs MCI+ve	MCI-ve vs AD	MCI+ve vs AD	
**PP**	p = 1.0	p = 0.06	p <0.001	p = 0.2	p <0.001	p = 0.09	
**PM**	p = 0.3	p <0.001	p <0.001	p = 0.002	p <0.001	p = 0.6	

**Table 4. 4MT Scores**. 4MT scores (scored out of 15) for all participants (top) and for MCI patients grouped according to CSF AD biomarker status (middle), with pairwise comparisons (bottom). HC = Healthy Controls; MCI = Mild Cognitive Impairment; AD = Alzheimer's Disease. Reproduced, with permission, from Moodley *et al*. (2015)^20^.


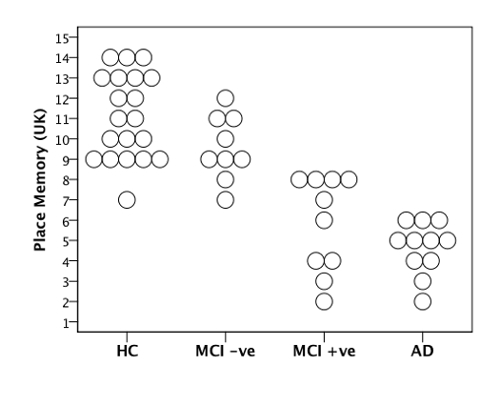
**Figure 2.4MT Scoresfor MCI Patients**. 4MT scores (scored out of 15) for MCI patients grouped by CSF AD biomarker status. Reproduced, with permission, from Moodley *et al*. (2015)^20^.

The ability of 4MT to differentiate between MCI patients with AD pathology (*i.e.*, MCI-ve and MCI+ve is illustrated by the area under the Receiver Operating Characteristics curve (AUC ROC) (**Figure 3**). Test performance was associated with an AUC of 0.93; PM scores of 8 or below were associated with 100% sensitivity and 78% specificity for differentiating MCI+ve from MCI-ve individuals.


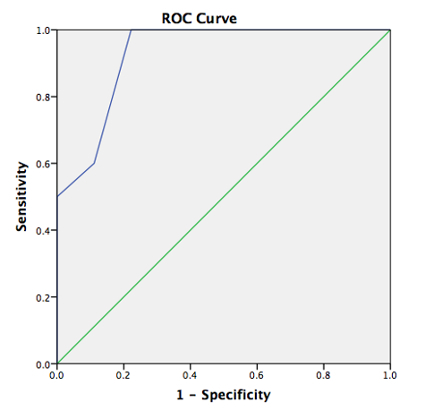
**Figure 3.ROC Curve.** ROC curve showing discrimination of MCI patients with and without biomarker evidence of AD. Area under ROC curve 0.93.

*Correlations between 4MT and quantitative MRI data* Partial correlations were undertaken for patients with MCI and AD-related dementia, corrected for age and total intracranial volume. After averaging between left and right hemispheres, significant associations were found between PM score and hippocampal volume (r = 0.42, p = 0.03, not surviving the corrected alpha threshold of 0.02), and between PM score and cortical thickness of the precuneus (r = 0.55, p = 0.003). No significant correlation between PM score and the cortical thickness of the posterior cingulate gyrus was observed (r = 0.19, p = 0.4). Scatterplots of these correlations are provided in **Figure 4**.


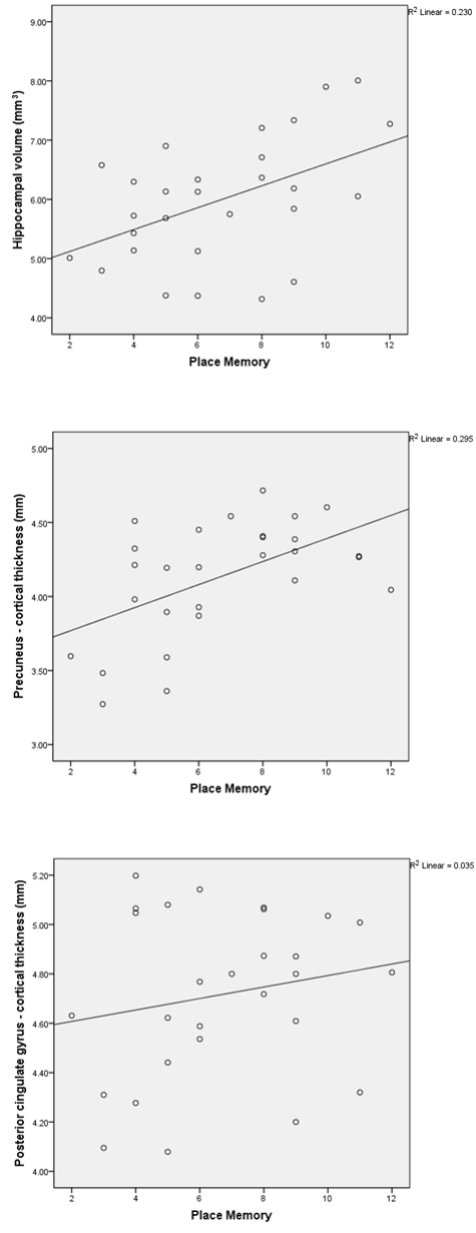
**Figure 4.Scatterplots Demonstrating Correlation With Structural MRI Data.** Scatterplots demonstrating correlation between 4MT score and hippocampal volume (top), cortical thickness of the precuneus (middle) and cortical thickness of the posterior cingulate gyrus (bottom) for all MCI and AD patients. Reproduced, with permission, from Moodley *et al*. (2015)^20^.

*Testing of 4MT stability and reliability* Psychometric properties of the 4MT were evaluated in a separate cohort of 41 healthy controls without symptoms of cognitive impairment. Participants were retested 7 and 28 d after initial testing. The effect size between the mean score at baseline and at 7 and 28 d was assessed using the Cohen's *d* statistic. The modest practice effect observed at 7 d (*d *= 0.35) was eliminated by 28 d (*d *= 0), indicating that there was no demonstrable practice effect by the latter interval.

A high degree of reliability was found between 4MT performance at baseline and retest. The average measure intraclass coefficient was 0.808 (95% CI 0.54 -0.918, F23, 23 = 5.96, p <0.01) and 0.641 (95% CI -0.115 - 0.862, F16, 16 = 2.49, p <0.05) at 7 and 28 d, respectively. The mean difference in test score was 0.71 ± 1.52 and 0 ± 2.24 at 7 and 28 d, respectively.

The stability and reliability of the 4MT in patient participants will be assessed in upcoming, larger scale studies.

## Discussion

The 4 Mountains Test (4MT) is a short test of allocentric spatial memory which is sensitive to predementia AD in patients exhibiting MCI. Following on from previous studies showing that this test can differentiate between AD-related and non-AD-related dementia^18, 19^, 4MT scores differed significantly between groups of MCI patients with and without CSF biomarker evidence of AD, who were otherwise matched in terms of demographics, symptom duration, premorbid IQ and performance on general neuropsychometric testing. Of particular note, there was no significant difference between the 2 MCI groups in terms of Rey Auditory Verbal Learning Test (RAVLT) performance. The RAVLT is a widely used test of episodic memory, considered to have high diagnostic sensitivity for early AD, cited as one of the cognitive tests suitable for use in diagnosing MCI due to AD^3^. The ability of 4MT testing to detect the presence of early AD is further illustrated by measures of test sensitivity and specificity. A 4MT score of 8/15 or below was associated with 100% diagnostic sensitivity and 78% specificity for the detection of early AD in MCI patients, as demonstrated by biomarker evidence of AD. The related area under the Receiver Operating Characteristic curve was 0.93.

4MT scores correlated with hippocampal volume (r = 0.42) and with the cortical thickness of the precuneus (r = 0.55), consistent with the viewpoint that allocentric spatial memory in humans is subserved by a functional network that encompasses the hippocampus and the precuneus. Task-free fMRI studies show that the hippocampus and precuneus represent highly interconnected hubs within a "default mode network" underpinning spatial and episodic memory^39, 40^ and the findings of this recent study are in keeping with the vulnerability of this network to early AD^41^.

Taken together, the results obtained from several studies involving patients with MCI and AD prove the principle that the 4MT is a sensitive test for predementia AD. The potential added value of this test is highlighted by observations in this recent work that neither hippocampal volume, considered to be a biomarker of AD, or testing of episodic memory with the RAVLT, were able to discriminate between MCI patients with and without evidence of underlying AD. Further larger scale studies involving the 4MT are currently in progress that will address the major limitation of this study that relates to its relatively small sample size. These include longitudinal studies that assess the ability of 4MT performance to predict conversion of MCI to dementia and longitudinal studies in asymptomatic elders to determine if impaired 4MT performance may herald subsequent AD-related cognitive decline. AD versus non-AD specificity studies will address the question of test-retest reliability in patients rather than controls, as described in this paper.

*Critical steps in test administration* The test is straightforward to administer but care must be taken to provide accurate instructions and practice. In particular, pilot work with the task suggests that the wording of instructions is important, as the allocentric (view-independent) nature of the recognition task can seem unfamiliar. Participants may have idiosyncratic interpretations of terms such as "scene", "image", "landscape" but they do understand the phrase "same place" to encompass alternative views. A critical step in the protocol is ensuring that this element of the instruction has been understood before progressing from practice to test items.

Results suggest that the test is selectively sensitive to hippocampal pathology but, in principle, performance can be also limited by patient cooperation, motivation and attention (as affected by depression or anxiety, for example), by their understanding of the instructions and potentially by damage to other brain regions involved in vision and allocentric spatial memory. It is important therefore, that test participants are motivated and attentive and that inclusio /exclusion criteria and accompanying measures are sufficient to exclude alternative explanations for poor performance.

*Test modification* The 4MT is now being used as an outcome measure for interventional studies of anti-AD drugs, applied to people prior to dementia onset. In view of the need for repeated testing, an alternate 15-item form of the test, matched for test difficulty with the 15-item test used in 3 studies, has already been generated, notwithstanding the good test-retest reliability of the current stimuli. Further difficulty-matched 15-item alternate-version tests are currently being developed in order to facilitate multiple repeat testing within future longitudinal studies and treatment trials.

Several modifications to the protocol described above can be adopted. In the published clinical studies, patients and controls were tested with a 15-item topographical memory test. For research purposes other versions of the task may be useful. Perceptual (sample image remains visible during matching) and non-spatial variants (in which participants match texture, lighting and weather conditions rather than spatial organization) of the task have been used for comparison with topographical memory^10^ which might enable detection of confounding motivational or perceptual issues. In nonclinical studies with exclusively healthy participants^21^ a more challenging 30-item topographical memory test may be used.

*Limitations of the technique* Successful application of the 4MT requires visual function that is sufficiently intact to perceive the test stimuli.

In conclusion, the 4MT has several operational advantages which would favor its use as a diagnostic test for pre-dementia AD in routine clinical practice. Specifically, its brevity, ease of administration and non-invasive nature allows it to be used in nonspecialist, as well as specialist, clinical settings. In view of these advantages, ongoing work is exploring the feasibility of applying this test to larger patient cohorts derived from community-based memory clinics and hospital clinics, with prolonged follow-up to determine the ability of the 4MT to predict conversion from MCI to dementia. The provision of the 4MT in electronic form will facilitate the planned widespread adoption of this test as a clinical diagnostic tool.

## Disclosures

The authors declare that they do have not competing financial interests.
